# Postoperative Pain Following Transoral Thyroidectomy via Vestibular Approach and Cervical Thyroidectomy: A Systematic Review and Meta-Analysis

**DOI:** 10.7759/cureus.59998

**Published:** 2024-05-09

**Authors:** Hyder Mirghani, Bandar Ahmed Alamrani, Fadi Olyan Alamrani, Mohammed Abdullah S Alasmari, Mohammed Ahmed I Albalawi, Hatem Hamad M Alquthami, Ali Ahmed Ali Alalawi, Omar Sabbah Alzamhari, Abdulaziz Nasser Albalawi, Mohammad Omar Aljabri, Turki Suleman Albalawi, Ahmed Mohammed Albalawi

**Affiliations:** 1 Department of Internal Medicine, University of Tabuk, Tabuk, SAU; 2 Department of Ear, Nose, and Throat (ENT), King Fahad Specialist Hospital, Tabuk, SAU; 3 College of Medicine, University of Tabuk, Tabuk, SAU; 4 Department of Internal Medicine, King Fahad Specialist Hospital, Tabuk, SAU; 5 Department of Emergency Medicine, King Khalid University Hospital, Tabuk, SAU

**Keywords:** complications of thyroidectomy, thyroidectomy, postoperative pain, conventional thyroidectomy, vestibular approach, transoral endoscopic thyroidectomy

## Abstract

Transoral endoscopic thyroidectomy via the vestibular approach (TOETVA) represents a minimally invasive alternative to traditional open thyroidectomy (OT). The objective of this systematic review and meta-analysis was to comprehensively analyze and compare postoperative pain outcomes between conventional open thyroidectomy (COT) and TOETVA. We conducted a systematic search across multiple databases, including PubMed, Medline, Elton B. Stephens Company (EBSCO), and Google Scholar, to identify cohorts and randomized trials comparing postoperative pain outcomes between patients undergoing transoral endoscopic thyroidectomy via the vestibular approach (TOETVA) and those undergoing conventional thyroidectomy. The search period spanned from the earliest available article up to January 15, 2022. Keywords such as "scarless thyroidectomy," "endoscopic transoral via vestibular thyroidectomy," "conventional thyroidectomy," "transcervical thyroidectomy," "postoperative pain," and "visual analog pain score" were utilized to retrieve relevant studies.

A total of 1,291 patients from 11 studies were included in our analysis, with 10 studies originating from Asia and one from Europe. Among these studies, seven were prospective, while four were retrospective. The primary outcome measure was postoperative pain. Various statistical tests were also performed for data analysis, including the Chi-square and random effects model. The Newcastle Ottawa Scale was used to assess the quality of studies. There was no significant statistical difference observed between the endoscopic transoral vestibular route and the conventional cervical approach in terms of visual analog scale (VAS) score, with an odds ratio of -0.37 and a 95% confidence interval ranging from -0.9 to 0.17. The overall effect had a P-value of 0.18. However, substantial heterogeneity was noted, with an I2 value for heterogeneity of 98% and a P-value for heterogeneity of less than 0.001. The Chi-square value was calculated as 364.02, and the main difference was 9. In comparison, TOETVA exhibited lower pain levels on the first day post-operation compared to conventional thyroidectomy, with an odds ratio of -1.36 and a 95% confidence interval ranging from -2.65 to -0.06. Transoral endoscopic thyroidectomy via the vestibular approach demonstrated superior outcomes compared to conventional thyroidectomy in terms of postoperative pain management on the first day following surgery. However, when considering overall pain management throughout the recovery period, no significant difference was observed between the two approaches. More extensive studies evaluating pain levels on the day of surgery and controlling for analgesic interventions are warranted.

## Introduction and background

Thyroid cancer is a type of cancer that affects the parenchymal cells of the thyroid gland [1]. It ranks fifth among all types of cancer among females in the United States, accounting for 1%-4% of all malignancies [2]. It has approximately a 3:1 female preponderance [3]. According to reports, the incidence of thyroid cancer has increased steadily on a global scale; in particular, the detection rate of papillary thyroid carcinoma (PTC) has surged by 240% over the past three decades [4,5]. It is hypothesized that this rise in incidence, which has been observed across all ethnicities and genders, is predominantly attributable to an upward trend in the rate of diagnostic imaging [6]. There are two main types of thyroid cancers: follicular and papillary carcinoma [4]. These two types comprise over 90% of well-differentiated thyroid cancers [4]. Additionally, thyroid disorders such as benign nodules and goiter are prevalent within the spectrum of thyroid conditions [5].

Conventional open thyroidectomy (COT) is the established procedure for treating thyroid cancer, namely, papillary thyroid carcinoma (PTC) or thyroid nodules [7]. The first groundbreaking classical open conventional cervical thyroidectomy was established in 1898 by Theodor Kocher [8]. It is the most common surgical procedure for thyroid problems, usually using an anterior cervical "necklace" incision [8]. While studies report successful outcomes, it is important to acknowledge that this surgical procedure has certain limitations [9]. For example, cervical thyroidectomy may pose risks such as inadvertent removal of parathyroid glands and damage to the recurrent laryngeal nerve, which studies suggest are increasingly common occurrences [9]. Postoperative pain, difficulty swallowing, post-perinatal adhesions, hoarseness of voice, and paresthesia during neck movement may also develop following a routine classical thyroidectomy [10]. Individuals who have an exposed neck surgical incision may suffer from psychological distress and issues with their self-image [11].

Due to these complications, there continues to be considerable interest in minimally invasive thyroidectomy procedures that bypass the cosmetic and surgical consequences [8]. There are approximately 20 techniques for thyroid surgery at present [12]. Two endoscopic procedures are currently favored, particularly transthoracic approaches and transaxillary approaches [13]. Although these methods result in minimal scarring, they require extensive dissection due to the scarcity of natural anatomical planes of access to the organ [14].

In addition to these methods, transoral endoscopic thyroidectomy via the vestibular approach (TOETVA) offers a promising alternative to traditional thyroidectomy. This technique involves minimal tissue manipulation, maintains anatomical integrity, results in no visible scars, reduces postoperative complications, and shortens hospitalization duration [15].

Multiple studies document that TOETVA is gaining popularity with surgeons due to its acceptable inclusion criteria, comparatively brief learning curve, and favorable safety profile [16,17]. Additionally, it is gaining favor with patients due to its reduced postoperative pain, superior cosmetic outcomes, and corresponding improvement in quality of life [18]. Six investigations conducted in Thailand or China were compared in one meta-analysis of results of open thyroidectomy via cervical approach and TOETVA [19]. With the exception of the increased volume of drainage and prolonged operative time observed in the TOETVA group, the study found no statistically significant differences between the two methodologies regarding a comparatively limited set of postoperative outcomes [19]. A previous meta-analysis conducted a comparison of outcomes between transoral robotic/endoscopic thyroidectomy and open thyroidectomy (OT), revealing that transoral approaches demonstrated similar outcomes to OT [20]. However, it is worth noting that endoscopic and robotic techniques generally exhibit distinct operative and safety outcomes [21].

Postoperative pain management is crucial in thyroid surgery. Until now, only a few studies compared TOETVA and conventional thyroidectomy regarding postoperative pain scores. This is the first meta-analysis to assess postoperative outcomes between TOETVA and conventional cervical thyroidectomy.

## Review

Materials and methods

Literature Search

The researchers conducted a comprehensive search across multiple databases, including Elton B. Stephens Company (EBSCO), Google Scholar, PubMed, and Medline. The search was confined to articles published up to January 2024. Keywords such as "visual analog pain score," "scarless thyroidectomy," "endoscopic transoral via vestibular thyroidectomy," "conventional thyroidectomy," "transcervical thyroidectomy," and "postoperative pain" were utilized. Out of 207 initially retrieved articles, 107 met the eligibility criteria. Out of the total, 23 full texts underwent screening, including 11 studies in the meta-analysis. Data cross-checking was performed by two authors, with any discrepancies resolved through consensus. This meta-analysis was conducted in adherence to the Preferred Reporting Items for Systematic Reviews and Meta-Analyses (PRISMA) guidelines.

Inclusion Criteria and Exclusion Criteria

The studies were included if they were prospective or retrospective, published in English, and compared postoperative pain outcomes among patients who underwent thyroidectomy via the endoscopic transoral vestibular route and conventional cervical approach.

Case-control studies, randomized trials, case reports, study protocols, expert opinions, editorials, and systematic reviews were excluded from the analysis. Additionally, studies involving thyroidectomy via alternative routes such as areolar, breast axillary, robotic thyroidectomy, and periauricular approaches were also excluded from consideration.

Outcome Measures

The main outcome measure was postoperative pain compared between conventional thyroidectomy and endoscopic transoral vestibular approach.

Data Extraction and Quality of Assessment

**Figure 1:**
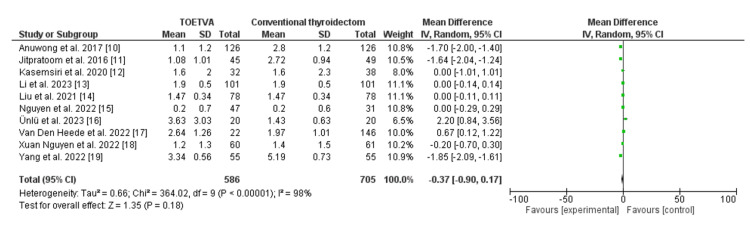
Comparison between transoral thyroidectomy via vestibular approach and transcervical thyroidectomy regarding postoperative pain TOETVA: transoral endoscopic thyroidectomy via the vestibular approach, SD: standard deviation, CI: confidence interval

A datasheet was used to extract the data regarding authors' names, publication years, patient numbers, study durations, patient selection criteria, and postoperative pain recorded using the visual analog scale (VAS). To assess the quality of studies, the Newcastle Ottawa Scale was used [22]. Four studies were found to be of good quality and seven with fair quality (Figure 1).

Data Analysis

All statistical analyses were performed using RevMan version 5.4 (The Cochrane Collaboration, Oxford, United Kingdom) software. Descriptive statistics were employed to summarize the characteristics of the included studies, such as patient demographics, pathology types, and surgical approaches. Odds ratios with corresponding 95% confidence intervals were calculated to determine the magnitude and direction of the effect size. The weighted average effect size (Z) was computed to assess the overall effect, with significance tests conducted using a P-value threshold of <0.05. The presence of heterogeneity among the studies was evaluated using the I2 statistic and Chi-square test, with a random effects model utilized due to substantial heterogeneity. Funnel plots were utilized to evaluate publication bias and lateralization, with significance defined as a P-value of less than 0.05.

Results

We analyzed data from 1,291 patients across 11 studies (Table 1) [13,16,23-31], with 10 originating from Asia and one published in Europe. The predominant demographic consisted of young females undergoing TOETVA procedures. Importantly, the pathology of thyroid disease was found to be the same between the two observed groups, although patients undergoing TOETVA tended to undergo more lobectomies (Table 2).

**Table 1:** Total number of patients included in 11 studies

Author	Total number of patients included (transoral vestibular approach thyroidectomy)	Total number of patients included (open thyroidectomy)
Anuwong et al. (2018) [16]	126 patients	126 patients
Jitpratoom et al. (2016) [23]	45 patients	49 patients
Kasemsiri et al. (2020) [24]	32 patients	38 patients
Li et al. (2023) [13]	101 patients	101 patients
Liu et al. (2021) [25]	78 patients	78 patients
Nguyen et al. (2022) [26]	47 patients	31 patients
Ünlü et al. (2023) [27]	20 patients	20 patients
Van Den Heede et al. (2022) [28]	22 patients	146 patients
Xuan Nguyen et al. (2022) [31]	60 patients	61 patients
Yang et al. (2022) [29]	55 patients	55 patients
Zhang et al. (2019) [30]	45 patients	41 patients

**Table 2:** Study characteristics

Author	Country	Age	Females	Benign nodules	Lobectomy (%)
Anuwong et al. (2018) [16]	Thailand	35.3±12.1 versus 35.3±12.1	92.2% versus 91.2%	Various disorders	58.1% versus 61.1%
Jitpratoom et al. (2016) [23]	Thailand	32.84±9.01 versus 32.79±9.53	88.9% versus 89.8%	All Grave's disease	Total thyroidectomy
Kasemsiri et al. (2020) [24]	Thailand	38.3±11.3 versus 46.7±10.9	100% versus 89.5%	96.9% versus 100%	Lobectomy in all
Li et al. (2023) [13]	China	32.2±7.0 versus 35.0±8.3	94.1% versus 94.1%	All thyroid carcinoma	Total thyroidectomy
Liu et al. (2021) [25]	China	Matched for age	50% versus 50%	All thyroid carcinoma	Total thyroidectomy
Nguyen et al. (2022) [26]	Vietnam	38±10.5 versus 52.5±13.4	97.9% versus 90.3%	100% in all	91.5% versus 74.2%
Ünlü et al. (2023) [27]	Turkey	42.9±9.7 versus 50.3±6.2	All females	65% versus 65%	35% versus 30%
Van Den Heede et al. (2022) [28]	France	40 versus 51	100% versus 76%	82% versus 63%	Hemithyroidectomy
Yang et al. (2022) [29]	China	41.35±5.86 versus 40.12±5.27	61.8% versus 69.1%	All thyroid carcinoma	Lobectomy in all
Zhang et al. (2019) [30]	China	33.7±10.2 versus 42.4±12.5	92% versus 77%	80% versus 60%	85% versus 12%
Xuan Nguyen et al. (2022) [31]	Vietnam	35.8±10.3 versus 46.9±11.5	90% versus 88.5%	22.3% versus 26.2%	76.6% versus 73.8%

The comparison between transoral thyroidectomy vestibular approach (TOETVA) and transcervical thyroidectomy (OT) in Table 3 reveals notable trends. Compared to patients undergoing OT, those undergoing TOETVA generally tend to be younger, with a slightly higher proportion of females. While clinical diagnoses vary between the two groups, with conditions such as Graves' disease and thyroid carcinoma reported, both approaches encompass a range of diagnoses. However, TOETVA often involves more lobectomies, whereas OT frequently entails total thyroidectomy. Although some studies show comparable ages and gender distributions, the overall findings suggest potential demographic and surgical differences between TOETVA and OT, warranting further investigation into their clinical implications and outcomes.

**Table 3:** Comparison of age, gender, clinical diagnosis, and surgical extent between transoral vestibular approach thyroidectomy and transcervical thyroidectomy TOETVA: transoral endoscopic thyroidectomy via the vestibular approach, OT: open thyroidectomy

Author	Age/years (TOETVA)	Age/years (OT)	Females% (TOETVA)	Females % (OT)	Clinical diagnosis (TOETVA)	Clinical diagnosis (OT)	Type of surgery (TOETVA)	Type of surgery (OT)
Anuwong et al. (2018) [16]	35.3±12.1	35.3±12.1	92.2%	91.2%	Various diagnosis	Various diagnosis	58.1% lobectomy	61.1% lobectomy
Jitpratoom et al. (2016) [23]	32.84±9.01	32.79±9.53	88.9%	89.8%	Graves' disease	All Graves' disease	Total thyroidectomy	Total thyroidectomy
Kasemsiri et al. (2020) [24]	38.3±11.3	46.7±10.9	100%	89.5%	96.% thyroid nodules	100% thyroid nodules	Lobectomy	Lobectomy
Li et al. (2023) [13]	32.2±7.0	35.0±8.3	94.1%	94.1%	All thyroid carcinoma	All thyroid carcinoma	Total thyroidectomy	Total thyroidectomy
Liu et al. (2021) [25]	Matched for age	Matched for age	50%	50%	All thyroid carcinoma	All thyroid carcinoma	Total thyroidectomy	Total thyroidectomy
Nguyen et al. (2022) [26]	38±10.5	52.5±13.4	97.9%	90.3%	100% thyroid nodules	100% thyroid nodules	91.5% lobectomy	74.2% lobectomy
Ünlü et al. (2023) [27]	42.9±9.7	50.3±6.2	10%	100%	65% thyroid nodules	65% thyroid nodule	35% lobectomy	0.30% lobectomy
Van Den Heede et al. (2022) [28]	40	51	100%	76%	82% thyroid nodules	63% thyroid nodules	Hemithyroidectomy	Hemithyroidectomy
Xuan Nguyen et al. (2022) [31]	35.8±10.3	46.9±11.5	90%	88.5%	22.3% thyroid nodules	26.2% thyroid nodules	76.6% lobectomy	73.8% lobectomy
Yang et al. (2022) [29]	41.35±5.86	40.12±5.27	61.8%	69.1%	All thyroid carcinoma	All thyroid carcinoma	Lobectomy	Lobectomy
Zhang et al. (2019) [30]	33.7±10.2	42.4±12.5	92%	77%	80% thyroid nodules	60% thyroid nodules	85% lobectomy	12% lobectomy

Table 4 illustrates the total pain analog score. Four studies reported the pain score on day 1 of surgery, and 10 studies assessed pain from day 2 and after. Seven studies were prospective, and four were retrospective. The study duration ranged from four to 64 months, and the type of analgesia and number of surgeons were stated in only four studies.

**Table 4:** Pain score, type and duration of the study, and analgesia used TOETVA: transoral endoscopic thyroidectomy via the vestibular approach, OT: open thyroidectomy

Author	Methods	Study duration	Total pain score/3 (TOETVA)	Total pain score/3 (OT)	Pain score/3 (day 1) (TOETVA)	Pain score/3 (day 1) (OT)	Analgesia used
Anuwong et al. (2018) [16]	Retrospective	28 months	1.1±1.2/126	2.8±1.2/126	2.1±1.9/126	4.9±1.8/126	Intravenous meperidine and acetaminophen
Jitpratoom et al. (2016) [23]	Retrospective	35 months	1.08±1.01/45	2.72±0.94/49	2.08±1.53	4.57±1.35	Not stated
Kasemsiri et al. (2020) [24]	Prospective	21 months	1.6±2/32	1.6±2.3/38	Not assessed	Not assessed	Not stated
Li et al. (2023) [13]	Retrospective	64 months	1.9±0.5/101	1.9±0.5/101	Not assessed	Not assessed	Not stated
Liu et al. (2021) [25]	Retrospective	36 months	1.47±0.34/78	1.47±0.34/78	Not assessed	Not assessed	Not stated
Nguyen et al. (2022) [26]	Prospective	24 months	0.2±0.7/47	0.2±0.6/31	2.9±0.7	3.3±0.6	Intravenous meperidine and acetaminophen
Ünlü et al. (2023) [27]	Prospective	4 months	3.63±3.03/20	1.43±0.63/20	3.13±3.17/20	1.17±1.53/20	Tramadol and paracetamol
Van Den Heede et al. (2022) [28]	Prospective	12 months	2.64±1.26/22	1.97±1.01/146	Not assessed	Not assessed	Not stated
Xuan Nguyen et al. (2022) [31]	Prospective	18 months	1.2±1.3/60	1.4±1.5/61	Not assessed	Not assessed	Not stated
Yang et al. (2022) [29]	Prospective	18 months	3.34±0.56/55	5.19±0.73/55	Not assessed	Not assessed	Not stated
Zhang et al. (2019) [30]	Prospective	7 months	Not assessed	Not assessed	0.5±0.3/45	2.69±1.7/41	Paracetamol, ketorolac, and morphine

Table 5 depicts the Newcastle Ottawa Scale of the included studies. Four studies were of good quality and seven with fair quality.

**Table 5:** Quality assessment

Study	Selection	Compatibility	Outcome	Overall
Anuwong et al. (2018) [16]	3	1	3	7
Kasemsiri et al. (2020) [24]	3	1	2	6
Li et al. (2023) [13]	3	1	3	6
Liu et al. (2021) [25]	3	1	2	6
Nguyen et al. (2022) [26]	3	1	2	6
Ünlü et al. (2023) [27]	3	2	3	8
Van Den Heede et al. (2022) [28]	3	1	2	6
Xuan Nguyen et al. (2022) [31]	3	1	2	6
Yan et al. (2022) [29]	3	1	3	7
Zhang et al. (2019) [30]	3	1	3	7
Jitpratoom et al. (2016) [23]	3	1	2	6

**Figure 2:**
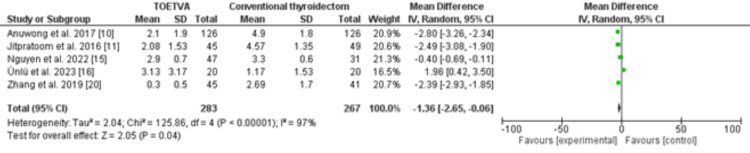
Comparison between transoral thyroidectomy via vestibular approach and transcervical thyroidectomy regarding postoperative pain (day 1) TOETVA: transoral endoscopic thyroidectomy via the vestibular approach, SD: standard deviation, CI: confidence interval

There was no significant statistical difference observed between the endoscopic transoral vestibular approach and the conventional cervical approach in terms of the visual analog score, with an odds ratio of -0.37 and a 95% confidence interval ranging from -0.9 to 0.17. The significance tests for the weighted average effect size produced a Z-score of 1.35, with an equivalent P-value for the overall effect of 0.18. However, substantial heterogeneity was noted, as indicated by an I2 value of 98% and a P-value for heterogeneity of less than 0.001. The Chi-square statistic was calculated to be 364.02, with a standard difference of 9. These findings are summarized in Figure 2.

**Figure 3:**
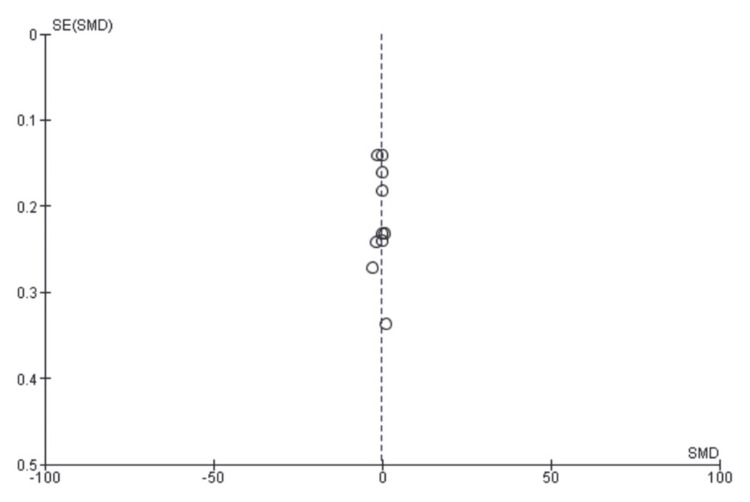
Comparison between transoral thyroidectomy via vestibular approach and transcervical thyroidectomy regarding postoperative pain (funnel and forest plots)

A sub-analysis revealed a lower pain score among patients who underwent the endoscopic transoral vestibular route compared to those who underwent the conventional cervical approach, with an odds ratio of -1.36 and a 95% confidence interval ranging from -2.65 to 0.06. The significance tests for the weighted average effect size resulted in a Z-score of 2.05, indicating a corresponding P-value of 0.04 for the overall effect. However, substantial heterogeneity was observed, as indicated by an I2 value of 97% and a P-value for heterogeneity of less than 0.001. The Chi-square statistic was calculated to be 125.86, with a standard difference of 4. These results are summarized in Figure 3.

Discussion

Summary of Findings

No significant statistical difference was evident between the endoscopic transoral vestibular approach and the conventional cervical approach in terms of the visual analog score, with an odds ratio of -0.37 and a 95% confidence interval ranging from -0.9 to 0.17. However, substantial heterogeneity was noted, as indicated by an I2 value of 98% and a P-value for heterogeneity of less than 0.001.

A sub-analysis revealed a lower pain score among patients who underwent the endoscopic transoral vestibular route compared to those who underwent the conventional cervical approach, with an odds ratio of -1.36 and a 95% confidence interval ranging from -2.65 to 0.06. However, substantial heterogeneity was observed, as indicated by an I2 value of 97% and a P-value for heterogeneity of less than 0.001.

With the popularization of minimally invasive penetration and smaller airframe trauma, transoral thyroidectomy is increasingly performed in thyroid surgery. TOETVA is a thyroidectomy technique that uses remote access and is associated with favorable postoperative outcomes and the absence of scarring [32]. According to a prior meta-analysis that contrasted TOETVA with conventional OT, TOETVA was found to be a viable and secure substitute procedure in terms of postoperative pain, blood loss, and drainage [32]. However, this meta-analysis contrasted the outcomes of OT and transoral thyroidectomy, in which both robotic and endoscopic approaches were incorporated. Our systematic review and meta-analysis are the first analysis of studies that compared the endoscopic vestibular approach and OT with the aim of revising the existing findings and investigating additional outcomes such as the occurrence of postoperative pain in patients. According to our research, transoral endoscopic thyroidectomy via the vestibular approach demonstrated superior outcomes compared to conventional thyroidectomy in terms of postoperative pain management on the first day following surgery. Importantly, TOETVA showed a lower pain score on day 1 of surgery. Our result implies that TOETVA may be an effective alternative approach to the conventional transcervical route with a better pain score. This finding supports the results of another meta-analysis that revealed that patients undergoing TOETVA experienced less postoperative pain than those undergoing OT, particularly until postoperative day (POD) 4. However, pain scores became comparable by POD 7 [33]. This difference in early postoperative pain may be attributed to the less painful nature of oral vestibule incisions compared to neck skin incisions in TOETVA. Oral wounds exhibit characteristics such as a reduced abundance of immune and profibrotic mediators, a higher proportion of bone marrow-derived cells, faster re-epithelialization, and more rapid fibroblast proliferation than dermal wounds. Consequently, oral mucosal healing occurs more swiftly and without scar tissue formation.

In contrast to our included study's findings in a comparative study between open thyroidectomy and TOETVA, the lower chin and lower lip, which act as TOETVA access sites, as well as the anterior neck area, where the incision is created for an open thyroidectomy, were evaluated independently for pain complaints [27]. Both patient groups received identical preoperative and postoperative analgesia protocols. As expected, the visual analog scale score in the chin and lower lip was notably higher in the TOETVA group in comparison with the open thyroidectomy group, gradually declining in both regions over time owing to the port entry areas and incision sites. In all groups, neck pain gradually diminished although it peaked in the second postoperative hour. Although the TOETVA group's VAS scores were only significantly higher in the second postoperative hour, no significant differences were observed in other comparisons. Notably, neck pain exhibited a significant decrease over time in both study groups.

One of our included studies by Zhang et al. [30] discovered that the TOETVA group had a reduced VAS score in the anterior neck and while swallowing during the first 24 hours postoperatively [31]. The study suggested that the reduced pain experienced in TOETVA compared to open surgery might be due to the higher density of pain receptors in the skin compared to the mucosa, as well as the lower density of pain receptors in the subplatysmal area. Furthermore, the reduced postoperative pain might be the result of the unique mobility features of the incision sites, which enable patients to control their discomfort more by stabilizing the inferior vestibular area in TOETVA.

Notably, recent research introduced a classification system for different mandibular jawlines, revealing varying impacts on TOETVA outcomes. Specifically, a Wang angle within the range of 80°to 110° demonstrated a greater reduction in pain compared to a B angle exceeding 110° or a C angle below 80° [23-34].

Overall, TOETVA emerges as a preferable option when factors such as cosmetic considerations and no pain outweigh concerns regarding cost, minimal infection risk, and extended operation duration. Furthermore, mastering proficiency in all forms of endoscopic thyroid surgeries poses a challenge for surgeons.

Study Strength

The strength of this study is that it is the first to compare TOETVA and conventional thyroidectomy regarding postoperative pain.

Study Limitations

There were several limitations to this research. Firstly, the study duration was very short, and only a limited number of studies were included in the current meta-analysis. Secondly, most of the studies were published in Asia, and only one study was from Europe. Lastly, there was a lack of uniformity in the administration and documentation of analgesia across studies, which introduces variability and complicates the interpretation of outcomes. Furthermore, the substantial heterogeneity observed is a major limitation.

## Conclusions

Transoral endoscopic thyroidectomy via the vestibular approach demonstrated superior outcomes compared to conventional thyroidectomy in terms of postoperative pain management on the first day following surgery. However, when considering overall pain management throughout the entire recovery period, there was no significant difference observed between the two approaches. Larger studies evaluating pain levels on the day of surgery and controlling for analgesic interventions are warranted.
